# Nuclear MAST4 Suppresses FOXO3 through Interaction with AKT3 and Induces Chemoresistance in Pancreatic Ductal Carcinoma

**DOI:** 10.3390/ijms25074056

**Published:** 2024-04-05

**Authors:** Rina Fujiwara-Tani, Takamitsu Sasaki, Ujjal Kumar Bhawal, Shiori Mori, Ruiko Ogata, Rika Sasaki, Ayaka Ikemoto, Shingo Kishi, Kiyomu Fujii, Hitoshi Ohmori, Masayuki Sho, Hiroki Kuniyasu

**Affiliations:** 1 Department of Molecular Pathology, Nara Medical University, 840 Shijo-cho, Kashihara 634-8521, Nara, Japan; takamitu@fc4.so-net.ne.jp (T.S.); m.0310.s.h5@gmail.com (S.M.); pkuma.og824@gmail.com (R.O.); a.ikemoto.0916@gmail.com (A.I.); nmu6429@yahoo.co.jp (S.K.); toto1999-dreamtheater2006-sms@nifty.com (K.F.); brahmus73@hotmail.com (H.O.); 2 Research Institute of Oral Science, Nihon University School of Dentistry at Matsudo, Matsudo 271-8587, Chiba, Japan; bhawal2002@yahoo.co.in; 3 Pathology Laboratory, Research Institute, Tokushukai Nozaki Hospital, 2-10-50 Tanigawa, Daito 574-0074, Osaka, Japan; 4 Department of Surgery, Nara Medical University, Kashihara 634-8522, Nara, Japan; m-sho@naramed-u.ac.jp

**Keywords:** pancreatic ductal adenocarcinoma, gemcitabine resistance, *MAST4*, stemness, FOXO3

## Abstract

Pancreatic ductal adenocarcinoma (PDAC) is highly malignant, with a 5-year survival rate of less than 10%. Furthermore, the acquisition of anticancer drug resistance makes PDAC treatment difficult. We established MIA-GEM cells, a PDAC cell line resistant to gemcitabine (GEM), a first-line anticancer drug, using the human PDAC cell line—MIA-PaCa-2. Microtubule-associated serine/threonine kinase-4 (*MAST4*) expression was increased in MIA-GEM cells compared with the parent cell line. Through inhibitor screening, dysregulated AKT signaling was identified in MIA-GEM cells with overexpression of AKT3. *MAST4* knockdown effectively suppressed AKT3 overexpression, and both *MAST4* and AKT3 translocation into the nucleus, phosphorylating forkhead box O3a (FOXO3) in MIA-GEM cells. Modulating FOXO3 target gene expression in these cells inhibited apoptosis while promoting stemness and proliferation. Notably, nuclear *MAST4* demonstrated higher expression in GEM-resistant PDAC cases compared with that in the GEM-sensitive cases. Elevated *MAST4* expression correlated with a poorer prognosis in PDAC. Consequently, nuclear *MAST4* emerges as a potential marker for GEM resistance and poor prognosis, representing a novel therapeutic target for PDAC.

## 1. Introduction

Pancreatic cancer is the third commonest cause of cancer-related deaths in Japan and the United States. In Japan, 44,000 people develop the disease, and 38,000 die annually [1], whereas in the United States, 64,050 people develop the disease and 50,550 die annually [2]. Pancreatic cancer incidence continues to increases and is predicted to become the second leading cause of cancer-related deaths in 10 years [3]. Pancreatic ductal adenocarcinoma (PDAC) is highly malignant, with distant metastasis in 50% of presenting cases and curative resection achieved in only 15–20% of cases [4,5]. PDAC has a poor prognosis, even with multidisciplinary treatment, with an overall 5-year survival rate of only 8.5% [1]. Consequently, anticancer drugs are becoming crucial. However, anticancer drug resistance frequently occurs during PDAC chemotherapy, making treatment difficult [6].

Resistance to gemcitabine (GEM), one of major anticancer drugs against PDAC, develops soon after its administration [6]. Cancer cell stemness significantly contributes to its resistance despite the multifactorial underlying mechanism [7]. In our previous study, we examined GEM-resistant PDAC cell lines induced by continuous GEM treatment and observed multiple drug resistance; however, we did not observe the induction of known resistance genes, such as multiple drug resistant gene or deoxycytidine kinase [8,9]. Furthermore, altered energy metabolism and enhanced stemness in these resistant cell lines contribute to anticancer drug resistance in PDAC [8,9].

*MAST4* is a microtubule-associated serine/threonine kinase family gene with unclear substrates, although a few have been reported, including sox9 (SRY-box9), ERM (erythromycin ribosome methylase), and Rhotekin [10,11,12]. *MAST4* mutations are associated with neurodevelopmental disorders, developmental delay, and infantile spasms [13]. *MAST4* maintains and enhances stemness, such as in sperm stem cells [14], suppresses mesenchymal stem cell differentiation, and promotes β-catenin nuclear translocation [15]. Some reports of its association with cancer exist. Estrogen signaling negatively regulates multiple myeloma-associated bone lesions through *MAST4*. The reason behind this is the regulation of the PI3-kinase and mTOR pathways by *MAST4* [16]. Underexpression of *MAST4* is associated with survival in breast cancer patients [17]. Moreover, suppression of *MAST4* was found to be correlated with treatment outcome in breast cancer [18].

Thus, to date, no common multifactorial genetic changes have been associated with GEM resistance acquisition in PDAC. Therefore, we established a GEM-resistant PDAC cell line and searched for genes responsible for the differences in gene expression profiles compared with those in the parent line.

## 2. Results

### 2.1. Establishment of GEM-Resistant PDAC Cells

**Figure 1:**
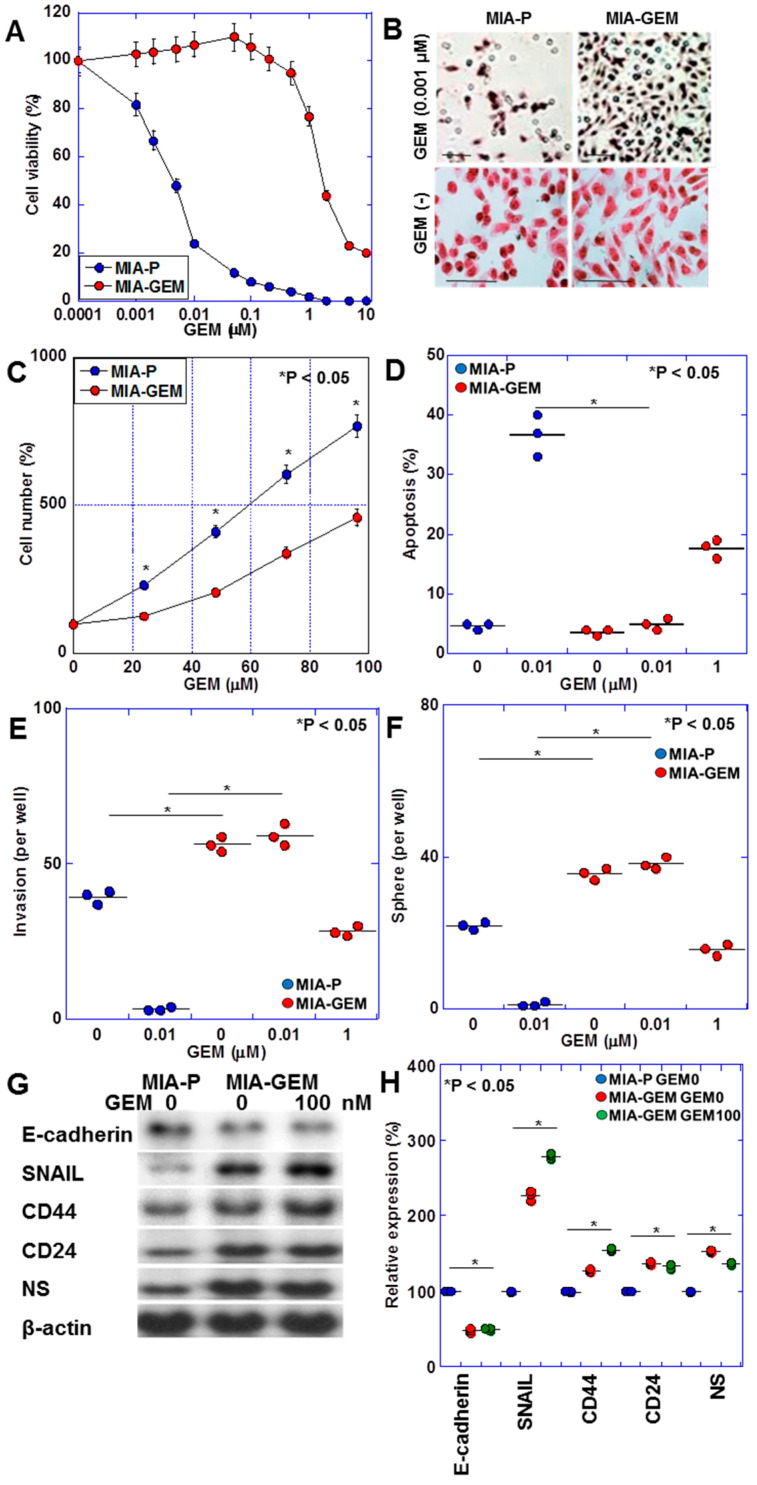
GEM-resistant cell line derived from human PDAC cell line—MIA-PaCa-2. (**A**) GEM sensitivity of MIA-P and MIA-GEM cells. (**B**–**G**) Characteristics of MIA-GEM cells in comparison with MIA-P cells: morphology (**B**), cell growth (**C**), GEM-induced apoptosis (**D**), in vitro invasion (**E**), sphere formation (**F**), and expression of stemness-related genes (**G**) with the semi-quantification (**H**). Scale bar, 50 μm. Error bars represent the standard deviations from three independent trials. Statistical differences were calculated using Student’s *t*-test. PDAC, pancreatic ductal adenocarcinoma; MIA-P, parental MIA-PaCa-2; MIA-GEM, gemcitabine-resistant MIA-PaCa-2; GEM, gemcitabine; NS, nucleostemin.

MIA-GEM cells were established by continuously treating MIA-PaCa-2 cells with low-dose GEM to induce resistance. GEM IC50 was increased to 3.5 μM, compared with 0.2 nM for the parent cell line (MIA-P cells) (Figure 1A). MIA-GEM cells were spindle-shaped compared with polygonal MIA-P cells (Figure 1B), and their proliferation rate was reduced to 62% of that of MIA-P cells (Figure 1C). Inducing apoptosis by GEM was reduced in MIA-GEM cells; however, the invasive ability of MIA-GEM cells was enhanced (Figure 1D,E). Sphere-forming ability of MIA-GEM cells was higher than that of MIA-P cells. Spheres of MIA-GEM cells showed resistance to GEM (Figure 1F). The expression of stemness markers, and E-cadherin expression decreased in MIA-GEM cells, while CD44, CD24, and nucleostemin (NS) expression increased (Figure 1G,H). Therefore, MIA-GEM cells acquired GEM resistance, enhanced stemness, and induced epithelial–mesenchymal transition.

### 2.2. Gene Expression Changes in GEM-Resistant PDAC Cells

**Figure 2:**
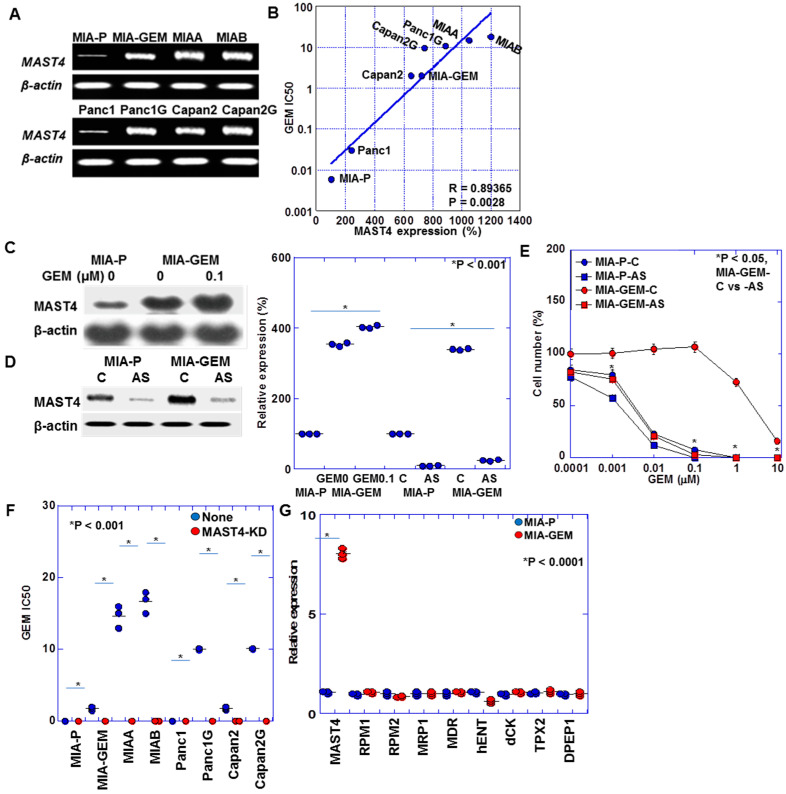
*MAST4* expression in PDAC cells. (**A**) *MAST4* expression in GEM-resistant human PDAC cells. (**B**) Relationship between *MAST4* expression and GEM IC50. (**C**) *MAST4* protein in GEM-treated MIA-GEM cells. (**D**) *MAST4* knockdown by AS. Right panel, semi-quantification of (**C**,**D**). (**E**) GEM sensitivity in *MAST4*-knocked down MIA-GEM cells. (**F**) GEM sensitivity in *MAST4*-knocked down PDAC cells. (**G**) Expression of known GEM-resistant genes. Error bar, standard deviation from three independent trials. Statistical differences were calculated using Student’s *t*-test. PDAC, pancreatic ductal adenocarcinoma; MIA-P, parental MIA-PaCa-2; MIA-GEM, GEM-resistant MIA-PaCa-2; GEM, gemcitabine; *MAST4*, microtubule-associated serine/threonine kinase family member 4; MIAA, MIAB; GEM-resistant MIA-PaCa-2 [8]; Panc1G, GEM-resistant Panc1 [8]; Capan2G, GEM-resistant Capan2 [8]; IC50, 50% inhibition concentration; C, sense S-oligonucleotide; AS, antisense S-oligonucleotide; RPM, NB-ARC domain-containing disease resistance protein; MRP, melittin-derived peptide; MDR, multiple drug resistance; hENT, human equilibrative nucleoside transporter; dCK, deoxycytidine kinase.

Table 1 summarizes the gene expression profile differences between MIA-GEM and MIA-P cells. A 7.18-fold increase in *MAST4* expression occurred in MIA-GEM cells. Furthermore, a 7.9-fold increase in *MAST4* expression was observed in MIA-GEM cells compared with that in MIA-P cells after reverse transcription polymerase chain reaction (Figure 2A). The other altered gene expression changes downregulated and upregulated patterns, which were 5–7-fold increased and 0.12–0.24-fold decreased. We investigated *MAST4*, which showed the most significant increase. Further examination of *MAST4* expression in other established GEM-resistant cell lines [8] revealed a *MAST4* overexpression in comparison to all parental cells. Increased *MAST4* expression correlated with increased IC50 after GEM sensitivity evaluation (Figure 2B). Furthermore, GEM treatment increased *MAST4* expression in the MIA-GEM cells (Figure 2C). The GEM sensitivity of MIA-GEM cells was equivalent to that of MIA-P cells after subsequent *MAST4* knockdown using antisense phosphorothioate (S)-oligodeoxynucleotide DNA (Figure 2D,E). When *MAST4* was knocked down in five GEM-resistant cell lines including MIA-G, GEM sensitivity increased to the level of the parent strain in all cell lines (Figure 2F). No significant increase in known GEM resistance-related gene expression in PDAC was observed in MIA-GEM cells (Figure 2G) [19,20,21,22].

**Figure 3:**
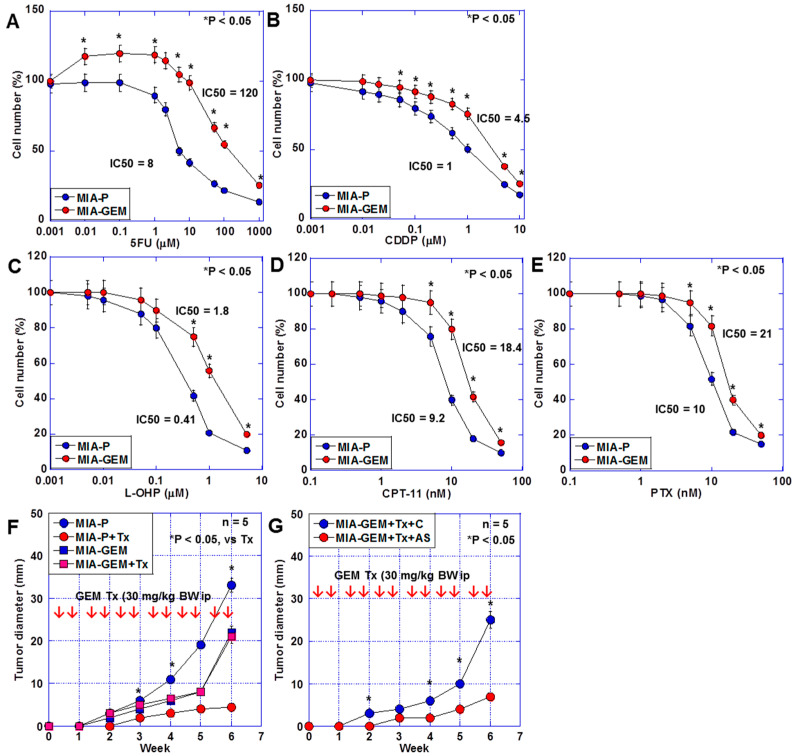
Multiple drug resistance in MIA-GEM cells. (**A**–**E**) Drug sensitivity of MIA-GEM cells to 5FU (**A**), CDDP, L-OHP, CPT-11, and PTX. (**B**). (**F**) Growth of subcutaneous tumors in nude mice. (**G**) Effect of *MAST4* knockdown on tumor growth. Red arrows indicate GEM administration (**F**,**G**). Error bar, standard deviation from three independent trials or five mice. Statistical differences were calculated using Student’s *t*-test. PDAC, pancreatic ductal adenocarcinoma; MIA-P, parental MIA-PaCa-2; MIA-GEM, GEM-resistant MIA-PaCa-2; GEM, gemcitabine; MAST4, microtubule-associated serine/threonine kinase family member 4; C, sense S oligonucleotide; AS, antisense S oligonucleotide; Tx, GEM treatment; 5FU, 5-fluorouracil; CDDP, cisplatin; L-OHP, oxaliplatin; CPT-11, irinotecan; PTX, paclitaxel.

The sensitivity of MIA-GEM cells to 5-fluorouracil (5FU), cisplatin (CDDP), oxaliplatin (L-OHP), irinotecan (CPT-11), and paclitaxel (PTX) was also lower than that of MIA-P cells (Figure 3A,B). Furthermore, the effect of GEM on MIA-GEM cells was reduced to one-sixth of that on MIA-P cells after examination using a nude mouse subcutaneous tumor model (Figure 3C). However, the GEM resistance level of MIA-GEM cells was reduced to that of MIA-P cells after *MAST4* knockdown (Figure 3D).

### 2.3. Identification of MAST4-Related Signals

**Figure 4:**
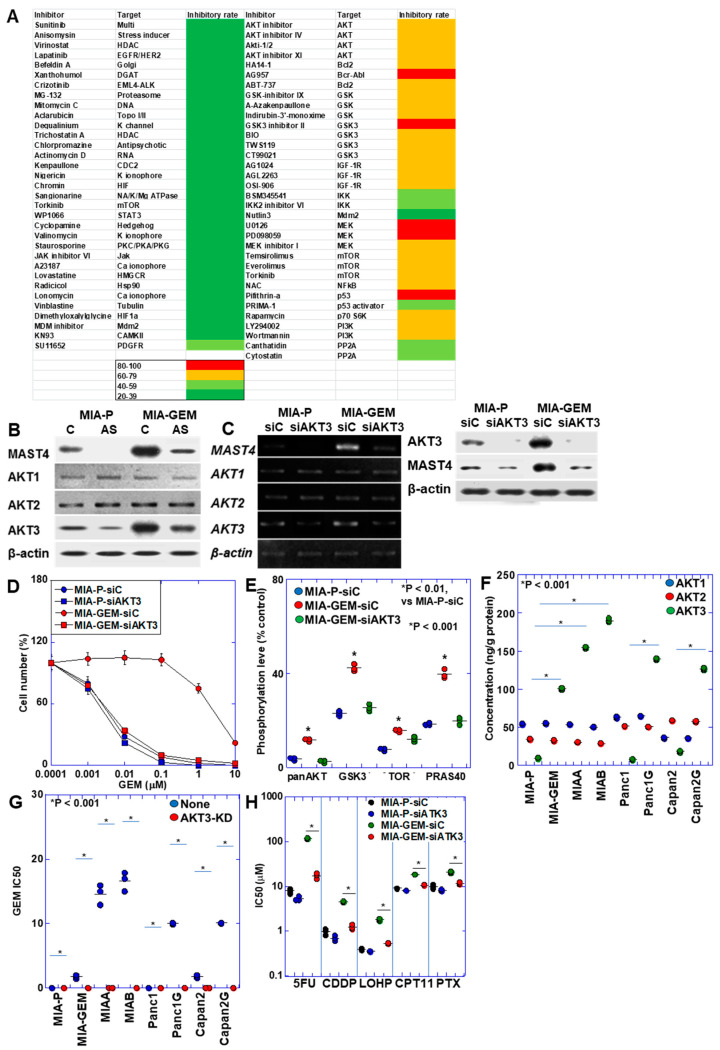
AKT3 in MIA-GEM cells. (**A**) Heat map of MIA-GEM cell-inhibitory effect of signal blockers. Inhibitors unrelated to AKT are shown in the left column, and AKT-related inhibitors are shown in the right column. Heatmap values indicate cell growth inhibition rate. (**B**) Effect of *MAST4* knockdown on protein expression of AKT family. (**C**) Effect of AKT3 knockdown on mRNA expression (upper) and protein (lower) of *MAST4* and AKT family. (**D**) GEM sensitivity in AKT3-knocked down MIA-GEM cells. (**E**) Phosphorylation levels of AKT3 signal proteins. (**F**) Protein levels of AKT family in GEM-resistant PDAC cells. (**G**) GEM sensitivity in AKT3-knocked down PDAC cells. (**H**) Effect of *AKT3* knockdown on other anti-cancer drugs. Error bar, standard deviation from three independent trials. Statistical differences were calculated using Student’s *t*-test. PDAC, pancreatic ductal adenocarcinoma; MIA-P, parental MIA-PaCa-2; MIA-GEM, GEM-resistant MIA-PaCa-2; MIAA, MIAB; GEM-resistant MIA-PaCa-2 [8]; Panc1G, GEM-resistant Panc1 [8]; Capan2G, GEM-resistant Capan2 [8]; GEM, gemcitabine; MAST4, microtubule-associated serine/threonine kinase family member 4; C, sense S-oligonucleotide; AS, antisense S-oligonucleotide; siC, control short interfering RNA; siAKT3, short interfering RNA to AKT3; GSK3β, glycogen synthase kinase 3β; TOR, target of rapamycin; PRAS40, proline-rich AKT substrate of 40-kDa; 5FU, 5-fluorouracil; CDDP, cisplatin; L-OHP, oxaliplatin; CPT-11, irinotecan; PTX, paclitaxel.

The results above suggest that *MAST4* is responsible for GEM resistance in MIA-GEM cells. Therefore, by screening various inhibitors, we extracted compounds with growth-inhibitory effects on MIA-GEM cells and analyzed *MAST4*-related signals (Figure 4A). Consequently, inhibitors of AKT and its related signaling pathways were identified. AKT exists in three isoforms; hence, we examined its protein levels (Figure 4B). AKT1 and AKT2 were similarly expressed in MIA-GEM and MIA-P cells, whereas AKT3 expression increased in MIA-GEM cells decreased by *MAST4* knockdown. Furthermore, *AKT3* expression was substantially suppressed after knockdown with siRNA, and *MAST4* expression decreased (Figure 4C). AKT3 knockdown decreased *MAST4* protein levels, restored the GEM sensitivity of MIA-GEM cells to a level similar to that of MIA-P cells (Figure 4C,D) and suppressed the phosphorylation level of AKT downstream signals, which were enhanced in MIA-GEM cells (Figure 4E). Next, we examined the protein levels of the AKT family in the five GEM-resistant cell lines (Figure 4F). No changes were observed in the levels of AKT1 and AKT2 in the resistant cells. In contrast, AKT3 protein levels increased in all resistant cells. Knockdown of *AKT3* increased GEM sensitivity in all cells (Figure 4G). In MIA-GEM cells, knockdown of *AKT3* increased the sensitivities to 5FU, CDDP, L-OHP, COT-11 and PTX (Figure 4H).

### 2.4. Regulation of MAST4 and AKT3 Expression

**Figure 5:**
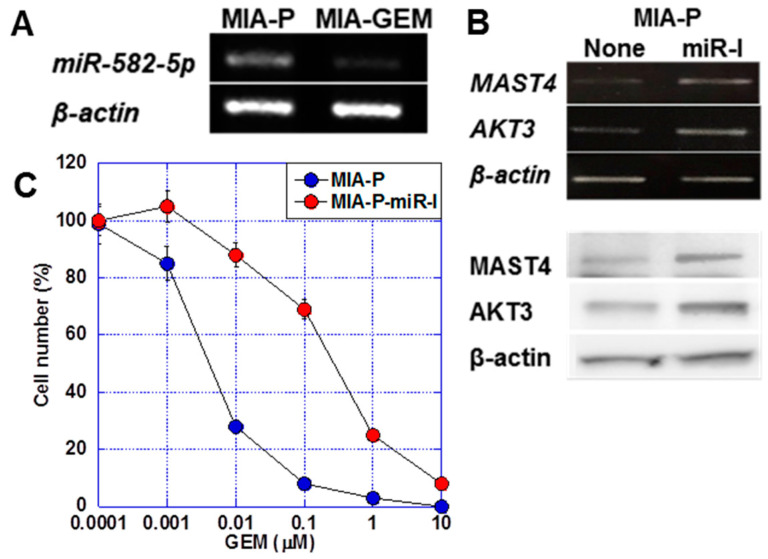
Role of miR-582-5p on *MAST4* and AKT3 expression. (**A**) Expression of *miR-582-5p* in MIA-GEM cells. (**B**) Effect of inhibition of *miR-582-5p* on expression of *MAST4* and AKT3. Upper, mRNA, lower, protein. (**C**) Effect of inhibition of *miR-582-5p* on GEM sensitivity in MIA-GEM cells. Error bar, standard deviation from three independent trials. Statistical differences were calculated using Student’s *t*-test. PDAC, pancreatic ductal adenocarcinoma; MIA-P, parental MIA-PaCa-2; MIA-GEM, GEM-resistant MIA-PaCa-2; GEM, gemcitabine; MAST4, microtubule-associated serine/threonine kinase family member 4; miR-I, miR-582-5p inhibitor.

*miR-582-5p* is a microRNA that regulates *AKT3* expression [23,24]. We observed a 6.0-fold increase in *miR-582-5p* expression in MIA-P cells compared with that in A-GEM cells (Figure 5A). The treatment of MIA-P cells with *miR-582-5p* inhibitor induced *MAST4* and *AKT3* expression at the mRNA and protein levels (Figure 5B). Furthermore, the treatment of MIA-P cells with *miR-582-5p* reduced the GEM sensitivity of MIA-P cells to a level comparable to that of MIA-GEM cells (Figure 5C).

### 2.5. Nuclear Translocation of MAST4 and AKT3

**Figure 6:**
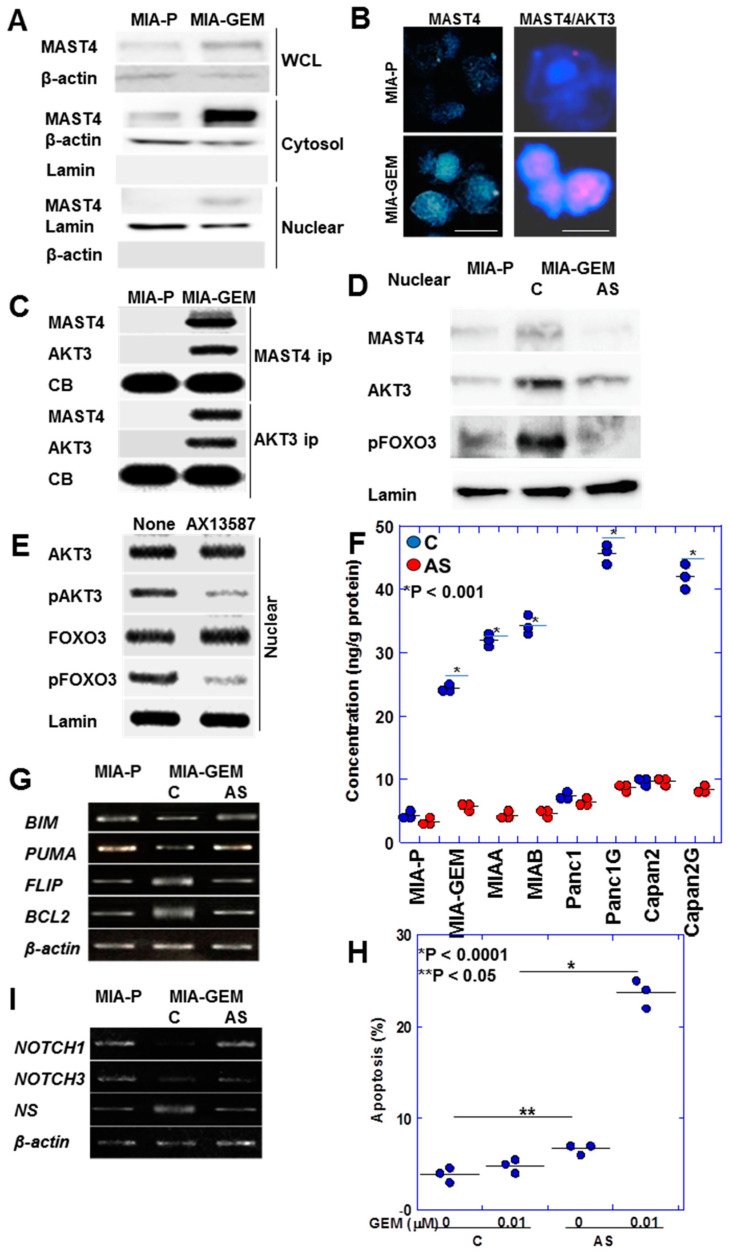
Intranuclear association between *MAST4* and AKT3. (**A**) Intracellular localization of MAST4. (**B**) Nuclear localization of *MAST4* (left) and proximity assay of *MAST4* and AKT3 (right). Scale bar, 20 μm. (**C**) Physical interaction between *MAST4* and AKT3 in the nuclei. (**D**) Phosphorylation of FOXO3 in the nuclei. (**E**) Effect of inhibition of *MAST4* kinase by AX13587 on AKT3 phosphorylation. (**F**) Effect of *MAST4* knockdown on phosphorylation of FOXO3 in the nuclei in PDAC cells. (**G**) Expression of apoptosis-associated genes of FOXO3 targets. (**H**) Effect of *MAST4* knockdown on GEM-induced apoptosis. (**I**) Expression of stemness-associated genes of FOXO3 targets. Error bar, standard deviation from three independent trials. Statistical differences were calculated using Student’s *t*-test. PDAC, pancreatic ductal adenocarcinoma; MIA-P, parental MIA-PaCa-2; MIA-GEM, GEM-resistant MIA-PaCa-2; MIAA, MIAB; GEM-resistant MIA-PaCa-2 [8]; Panc1G, GEM-resistant Panc1 [8]; Capan2G, GEM-resistant Capan2 [8]; GEM, gemcitabine; MAST4, microtubule associated serine/threonine kinase family member 4; WCL, whole cell lysate; cytosol, cytosol fraction; nuclear, nuclear fraction; ip, immunoprecipitant, C, sense S-oligonucleotide; AS, antisense S-oligonucleotide; CB, Coomassie blue; FOXO3, forkhead box protein O3; pFOX3, phosphorylated FOXO3; pAKT3, phosphorylated AKT3; BIM, BCL2 like 11; PUMA, p53 upregulated modulator of apoptosis; FLIP, Fas-associated death domain-like interleukin-1-converting enzyme-like inhibitory protein; BCL2, B cell lymphoma 2; NS, nucleostemin.

*MAST4* was intracellularly localized at a low cytoplasmic level in MIA-P cells; however, in MIA-GEM cells, it showed a substantial intracellular increase and was translocated into the nuclei (Figure 6A). Immunostaining revealed *MAST4* overexpression and nuclear translocation in MIA-GEM cells (Figure 6B). In addition, the Duolink^®^ proximity ligation assay revealed that *MAST4* and AKT3 were proximal in the nuclei (Figure 6B). Furthermore, in MIA-GEM cells, *MAST4* and AKT3 binding in the nucleus was confirmed via immunoprecipitation (Figure 6C). Investigating nuclear proteins revealed that FOXO3 (forkhead box O3a), an AKT3 downstream signal, was inactivated with a 4.5-fold increase in phosphorylation levels in MIA-GEM cells. Phosphorylated FOXO3, *MAST4*, and AKT3 levels were reduced in the nucleus after *MAST4* knockdown (Figure 6D). Furthermore, the treatment of MIA-GEM cells with the *MAST4* kinase inhibitor—AX13587—[25] inhibited AKT3 and FOXO3 phosphorylation (Figure 6E). This suggests that FOXO3 induces GEM resistance via nuclear *MAST4*/AKT3. To confirm this relationship, we examined nuclear pFoxO3 protein levels in five GEM-resistant PDAC cells (Figure 6F). Nuclear pFoxO3 protein was increased in all resistant cells and decreased by *MAST4* knockdown. Therefore, we examined the expression of apoptosis-related FOXO3 target genes (Figure 6G). MIA-GEM cells had decreased expression of BCL2 like 11 (*BIM*) and p53 upregulated modulator of apoptosis (*PUMA*) (pro-apoptotic) and increased expression of B-cell lymphoma 2 (*BCL2* and Fas-associated death domain-like interleukin-1-converting enzyme-like inhibitory protein (*FLIP*) (anti-apoptotic), respectively, compared to that in MIA-P cells; however, these effects ended after *MAST4* knockdown. *MAST4* knockdown also enhanced GEM-induced apoptosis (Figure 6H). Furthermore, we examined stemness-related gene expression among FOXO3 target genes (Figure 6I) owing to its association with dormant stemness maintenance [26,27]. *NOTCH1* and *NOTCH3* expression decreased in MIA-GEM cells compared with that in MIA-P cells but increased with *MAST4* knockdown. Concurrently, the expression of nucleostemin (*NS*), a stemness with proliferation marker [28], decreased with *MAST4* knockdown.

### 2.6. Role of MAST4 in PDAC Cases

**Figure 7:**
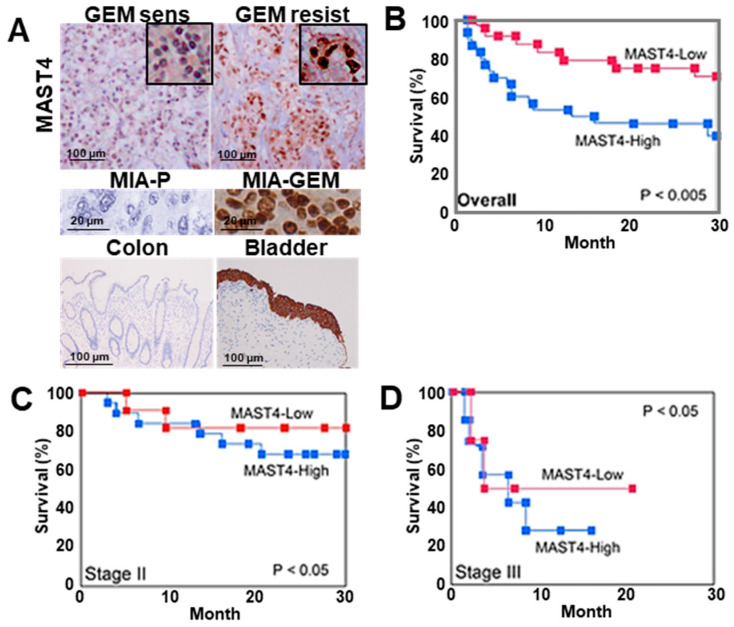
*MAST4* expression in human PDAC cases. (**A**) Immunohistochemical detection of *MAST4* in the nuclei of PDAC cases. Insets show high magnification. Middle panels, *MAST4* immunohistochemistry images in mouse tumors of MIA-P (negative control) and MIA-GEM (positive control). Lower panels, *MAST4* immunohistochemistry images in colon mucosa (negative control) and bladder urothelium (positive control). (**B**–**D**) Survivals between MAST4-high and -low cases; overall (**B**), stage II (**C**), and stage III (**D**). The MAST4-high and MAST4-low groups were divided based on the median *MAST4* expression. Survival curves were calculated using the Kaplan–Meier method. Statistical differences were calculated using the log-rank test. PDAC, pancreatic ductal adenocarcinoma; MIA-P, parental MIA-PaCa-2; MIA-GEM, GEM-resistant MIA-PaCa-2; GEM, gemcitabine; *MAST4*, microtubule-associated serine/threonine kinase family member 4.

We examined nuclear *MAST4* expression in 91 PDAC samples using immunostaining, which revealed distinct nuclear staining (Figure 7A). Immunocytochemistry images of MIA-P and MIA-GEM are shown as negative control and positive control, respectively. Furthermore, the colon mucosal epithelium and bladder urothelial epithelium are shown as negative control and positive control, respectively [29]. Of note, *MAST4* expression is high in the bladder urothelium, but positive in the cytoplasm and negative in the nucleus. This indicates that nuclear *MAST4* in PDAC has an abnormal subcellular distribution. The nuclear *MAST4* labeling index is correlated with differentiation grade, T factor, N factor, and PDAC stage (Table 2). Furthermore, significantly higher nuclear *MAST4* labeling was observed in chemoresistant cases (Table 2). Chemotherapy was administered to 88 patients excluding stage I, and *MAST4* was significantly expressed in progressive disease (PD) patients. In 43 cases that received GEM alone treatment, higher nuclear *MAST4* labeling was observed in resistant cases than in susceptible cases (Table 2 and Figure 7A). The 27 cases with known prognosis were divided into two groups based on the median nuclear *MAST4* expression. The clinicopathological parameters of each group are shown in Table 3. There were no differences between the two groups in parameters other than RECIST, while RECIST was worse in the MAST4-H group. In multivariate analysis, *MAST4* showed the most significant correlation with prognosis (Table 4). In addition, high nuclear *MAST4* indicated a significantly worse prognosis in all cases at stage II and stage III (Figure 7B–D).

## 3. Discussion

GEM-resistant MIA-GEM cells had enhanced stemness and anti-apoptotic survival, which also provide resistance to 5FU and CDDP. *MAST4* expression was higher in MIA-GEM cells than in MIA-P cells. *MAST4* translocated into the nucleus, bound to AKT3 in the nucleus, inactivated the AKT3 downstream signal, FOXO3, suppressed apoptosis, and promoted stemness with proliferation.

Our study revealed that AKT3 exists in the nucleus bound to *MAST4*. Nuclear translocation of AKT is dependent on PI3K phosphorylation [30,31]. AKT counteracts apoptosis by blocking caspase-activated DNase after translocation to the nucleus and is involved in cell cycle progression control, cell differentiation, mRNA transport, DNA repair, and tumorigenesis [32]. AKT also promotes stemness, sphere formation, aldehyde dehydrogenase activity, and increased stemness marker expression [33].

FOXO3, a member of the FOXO family located downstream of the PI3K/AKT pathway, binds to the DNA consensus sequence as a transcription factor [34]. FOXO3 promotes apoptosis by upregulating the pro-apoptotic factors—BIM and PUMA, and downregulating the anti-apoptotic factors—FLIP and BCL2 [35,36,37]. Therefore, it acts as a tumor suppressor gene in cancer [38]. Nuclear FOXO3 phosphorylation is correlated with high-grade and recurrent bladder cancer [39]. FOXO3 suppresses cancer stem cell propagation and improves disease prognosis [40]. In addition, FOXO3 maintains dormant stemness [26,27] and suppresses cancer stem cells [41]. Our results suggest that nuclear *MAST4*/AKT3 inactivates FOXO3, increases apoptosis resistance, and induces stemness with proliferation, resulting in GEM resistance.

*MAST4* has a single C-terminus PDZ domain [42] through which it forms a protein complex [43]. It also promotes interactions between bound proteins [44,45,46]. These findings suggest that MAST4 may enable FOXO3 phosphorylation by AKT3. MAST1-3 bind to PTEN [47] through the PDZ domain [48,49]; however, to our knowledge, our study is the first to report binding between *MAST4* and AKT3. *MAST4* also binds PTEN in multiple myeloma [16]. These findings suggest a close relationship between *MAST4* and PI3K/AKT signaling. However, a detailed examination of the binding possibility and three-dimensional structures of *MAST4*, AKT3, and FOXO3 is required. Knocking down one of *MAST4* and AKT3 reduces the expression of the other, suggesting the possibility of a feedback loop. This also requires future consideration.

We added knock down of the MIA-P. It is a good idea to introduce the *MAST4* vector into MIA-P and create an overexpression model, and this is a topic for future research. In our data, a detailed examination of the mechanism of *MAST4* relies on the comparison of MIA-P and MIA-GEM. Several examinations are also performed in the other six cell lines. In the future, multiple mechanistic analyses will be necessary for other resistant cell lines. Among the cases we analyzed, all at stage II-IV received some form of chemotherapy. Cases other than GEM alone are also receiving chemotherapy, and *MAST4* overexpression induces drug resistance to 5FU, L-OHP, PTX, and COT-11, which are used in these cases. Therefore, it is difficult to establish a complete control cohort without the *MAST4* effect in our data. Future analysis through prospective studies is required. 

Our data revealed that nuclear *MAST4* is highly expressed in GEM-resistant PDAC cells and clinical GEM-resistant PDAC cases, highlighting its importance in PDAC GEM resistance. Nuclear *MAST4* is associated with disease progression and poor prognosis owing to stemness promotion by *MAST4*/AKT3/FOXO3. Our data corroborate nuclear *MAST4* as a predictive marker for GEM resistance. *MAST4* expression was induced by GEM in a concentration-dependent manner, suggesting that nuclear translocation was accelerated during GEM chemotherapy. However, obtaining cancer tissues from PDAC is clinically challenging, and developing more clinically convenient markers, such as blood *MAST4*, is necessary. The *MAST4* inhibitor—AX13587—inhibited the nuclear phosphorylation of AKT3 and FOXO3. This suggests that targeting *MAST4* may be effective in overcoming GEM resistance. AX13587 also inhibits JNK [25]. Therefore, developing an inhibitor with higher specificity for *MAST4* that is applied clinically is recommended.

## 4. Materials and Methods

### 4.1. Cell Lines

The MIA-PaCa-2 human PDAC cell line was purchased from Dainihon Pharmaceutical Co. (Tokyo, Japan). PANC-1 and Capan-2 cells were obtained from the American Type Culture Collection. Cells were cultured in Dulbecco’s modified Eagle’s medium supplemented with 10% fetal bovine serum at 37 °C in 5% CO_2_. GEM-resistant MIA-GEM, a MIAA, MIAB, PANC1G, and Capan2G cells were established by continuous treatment with GEM (1 μM, Sigma-Aldrich Inc., St. Louis, MO, USA) and CoCl2 (150 μM, Wako, Osaka, Japan), exceeding 40 passages. [8]. These cell lines are passaged to ensure that MIA-P cells and MIA-GEM cells have the same passage. Additionally, *miR-873* inhibitors (Invitrogen, Carlsbad, CA, USA) and AX13587 (LookChem, Zhejiang, China) were purchased. We performed all experiments in triplicate. For cell assays, 1 x 10^5^ cells were seeded on 6-well plate and treated for 48 h. The identity of the resistant cell lines and the parent cell lines was confirmed by an STR profiling service (Wako).

### 4.2. Cell Growth and Apoptosis

We assessed cell growth using the 3-(4,5-Dimethylthiazol-2-yl)-5-(3-Carboxymethoxyphenyl)-2-(4-Sulfophenyl)-2 H-tetrazolium (MTS) assay [9]. MTS assays were performed using a CellTiter 96 Aqueous One Solution Cell Proliferation Assay kit (Promega, Madison, WI, USA). Generally, cells (1 × 10^6^) were seeded on 6-well plate and treated for 48 h. The plates were read using a Multiskan FC microplate photometer (Thermo Fisher Scientific Inc, Tokyo, Japan) at a wavelength of 490 nm. Cells cultured with oligonucleotides were used as controls in the MTS assay. Apoptotic cells were stained with Hoechst 33343 dye (Dojindo, Kumamoto, Japan), and stained cells were counted from 1000 cells using a KEYENCE all-in-one microscope (Osaka, Japan).

### 4.3. Sphere Assay

We seeded 1000 cells per well on uncoated bacteriological 35-mm dishes (Corning Inc., Corning, NY, USA) in 3D Tumorsphere Medium XF (Sigma-Aldrich). Digital images of the spheres were subsequently captured and measured using a BZ-X710 all-in-one fluorescence microscope (KEYENCE) and an NIH ImageJ software (version 1.52, Bethesda, MD, USA), respectively, after 7 d. We performed all experiments in triplicate.

### 4.4. Chamber Invasion Assay

A modified Boyden chamber assay was performed to examine the in vitro invasive ability of the PDAC cells [50]. The filters were carefully removed from the inserts, stained with hematoxylin for 10 min, and mounted on microscope slides after incubation at 37 °C for 24 h. The number of stained cells in each insert was counted at 100× magnification. Invasive activity was quantified by calculating the average number of cells per insert well. We performed all experiments in triplicate.

### 4.5. Mouse Models

Four-week-old male Slc-nu/nu BALB/c mice (SLC Japan, Shizuoka, Japan) were maintained in accordance with institutional guidelines approved by the Committee for Animal Experimentation of Nara Medical University and the current regulations and standards established by the Ministry of Health, Labor, and Welfare (approval number 11716, 20 July 2016). MIA-P or MIA-GEM cells (1 × 10^7^) in 100 mL HBSS (Wako) were mixed with 100 mL of Matrigel solution (#356234, Corning Inc., Corning, NY, USA) and inoculated into mouse scapular tissue subcutaneously. [51]. Cells were pretreated with 3 µM of antisense or control S-ODN (Table 5) for 3 d. Thereafter, the mice were treated with GEM (30 mg/kg body weight) intraperitoneally, twice weekly. In each treatment, 5 mice were used. Tumor sized was measured with calipers once a week.

### 4.6. Antisense Phosphorothioate(S)-Oligodeoxynucleotide Assay

An 18-mer S-ODN targeting *MAST4* was synthesized and purified using reverse-phase high-performance liquid chromatography (Espec Oligo Service, Tsukuba, Japan) (Table 4). Each cell line was pretreated with 3 µM of antisense or control S-ODN for 2 d with medium exchange. Subsequently, the cells were used for further experiments [50].

### 4.7. mRNA Profiling

According to the manufacturer’s instructions, total RNA was extracted from MIA-P and MIA-GEM cells using TRI Reagent (Molecular Research Center, Inc., Cincinnati, OH, USA). Subsequently, mRNA profiling from the RNA was performed using a DNA array service (Filgen Inc., Nagoya, Japan). mRNA quality control revealed that OD280/260 ratio > 1.8, OD230 > 1.6, and S28/S18 ratio > 2.0. In the service, materials were analyzed using GeneChipTM Arrays (Applied Biosystems, Waltham, MA, USA). Data were analyzed using Transcriptome Analysis Console software (version 4.0.1.36, Applied Biosystems), from which genes with gene expression of more than 2-fold and less than 1/2-fold and whose ANOVA *p*-value was less than 0.05 were isolated.

### 4.8. Inhibitor Assay

A SCADS inhibitor kit (https://www.molpro.jp/explore/library/#hyoujun, accessed on 24 May 2018) was used for inhibitor assays. The kit was kindly provided by the Screening Committee of Anticancer Drugs, supported by a Grant-in-Aid for Scientific Research on Innovative Areas, Scientific Support Programs for Cancer Research, from the Ministry of Education, Culture, Sports, Science, and Technology, Japan.

### 4.9. Western Blotting

Whole-cell lysates were prepared using Radio-Immunoprecipitation Assay buffer supplemented with 0.1% sodium dodecyl sulfate (SDS) (Thermo Fisher) [52]. A Minute Cytoplasmic and Nuclear Extraction Kit (Invent, Biotechnologies, Inc., Bloomington, IN, USA) was used to extract the nuclear and cytosolic fractions. Protein assays were performed using a Protein Assay Rapid Kit (Wako). Lysates were separated using 7.5% or 10.0% SDS-polyacrylamide gel electrophoresis and transferred to nitrocellulose membranes. The membranes were inoculated with primary antibodies (Table 5) and subsequently incubated with a polyclonal rabbit/mouse anti-IgG antibody (DAKO, Glastrup, Denmark) at room temperature for 2 h. β-Actin was used as a loading control. Immune complexes were visualized using Fusion Solo (M&S Instruments, Osaka, Japan). Images were captured on a computer, and the signal strength was measured using the NIH ImageJ software.

### 4.10. Immunoprecipitation

Immunoprecipitation was performed using a previously described method [53]. Lysates were pre-cleaned in lysis buffer containing protein A/G agarose (Santa Cruz Biotechnology, Santa Cruz, CA, USA) for 1 h at 4 °C and subsequently centrifuged. The supernatants were inoculated with antibodies to AKT3 or *MAST4* (Table 4) and protein A/G agarose and incubated for 1.5 h at 4 °C. Centrifugation precipitates were washed three times with wash buffer and solubilized in 4× Laemmli Sample Buffer (Bio-Rad, Hercules, CA, USA) and 2-mercaptoethanol (Sigma-Aldrich). Thereafter, immunoblotting was performed using appropriate antibodies (Table 5).

### 4.11. Reverse Transcription–Polymerase Chain Reaction (RT–PCR)

RT–PCR was performed with 0.5 µg total RNA extracted from the three cell lines using an RNeasy kit (Qiagen, Germantown, MD, USA) to assess human and murine mRNA expression. The primer sets used are listed in Table 5 and were synthesized using Sigma Genosys (St. Louis, MO, USA). PCR products were electrophoresed on a 2% agarose gel and stained with ethidium bromide. *β*-Actin mRNA was amplified and used as the internal control.

### 4.12. Small Interfering RNA

Stealth-select RNAi interference (siRNA) targeting human *AKT3* was purchased from Sigma-Aldrich. AllStars Negative Control siRNA (Qiagen) was used as a control. The cells were transfected with 10 nM siRNA using Lipofectamine 3000 (Thermo Fisher) according to the manufacturer’s recommendations.

### 4.13. Duolink^®^ Proximity Ligation Assay

The assay was performed following the manufacturer’s instructions. The following is a brief description: MIA-GEM or MIA-P cells (1 × 10^5^) were seeded and incubated for 24 h on Nunc chamber slides (Thermo Fisher), and then incubated with Duolink^®^ (Sigma-Aldrich) Blocking Solution for 24 h and 60 min at 37 °C, respectively. Anti-AKT3 (mouse monoclonal, 0.5 μg/mL, Santa Cruz) and anti-MAST4 antibodies (rabbit polyclonal, 0.5 μg/mL, Merck, Tokyo, Japan) were incubated with the Duolink^®^ Antibody Diluent. The antibody mixture (40 μL) was added to the cell plate after removing the blocking solution and incubated at 37 °C for 2 h. Subsequently, MINUS and PLUS probe solutions (8 μL each) were added, and the plate was incubated at 37 °C for 1 h. A mixture of ligase and ligation buffer (40 μL) was added to the plate, which was subsequently incubated at 37 °C for 30 min after removing the probe solution and washing. Thereafter, a mixture of polymerase and amplification buffer (40 μL) was added, and the plate was incubated at 37 °C for 100 min. The cell plates were subsequently mounted with Duolink^®^ In Situ Mounting Media containing 4’,6-diamidino-2-phenylindole. Finally, the cells were observed under a BZ-X710 microscope (KEYENCE).

### 4.14. Enzyme-Linked Immunosorbent Assay (ELISA) and Fluorometric Assay

Whole-cell lysates and a nuclear fraction were prepared as described above. Protein assays were performed using a Protein Assay Rapid Kit (Wako Pure Chemical Corporation, Osaka, Japan). Using the extracted proteins, an ELISA kit was used to measure the target proteins according to the manufacturer’s instructions. The kits that were used are listed in Table 5.

### 4.15. Patients

Ninety-one patients with PDAC, diagnosed and operated on at Nara Medical University Hospital, were randomly selected, and 27 were followed up to investigate survival. Table 2 summarizes the basic patient information. Sample anonymization was performed prior to analysis to ensure strict privacy protection (unlinkable anonymization) because written informed consent was not obtained. All procedures were performed in accordance with the Ethical Guidelines for Human Genome/Gene Research enacted by the Japanese Government and were approved by the Ethics Committee of Nara Medical University (Approval Number 937, 10 October 2010). For survival analysis, We divided MAST4-high and MAST4-low groups based on the median *MAST4* expression. 

### 4.16. Immunohistochemistry

Consecutive 4-μm sections were immunohistochemically stained using the immunoperoxidase technique [51]. We used 0.2 µg/mL of anti-MAST4, anti-AKT3 (Table 5), and secondary antibodies (Medical and Biological Laboratories, Nagoya, Japan). Tissue sections were color-developed using diaminobenzidine hydrochloride (DAKO) and counterstained with Meyer’s hematoxylin (Sigma-Aldrich). Cells with 1000 nuclei immunoreactions were counted to determine the labeling index (%).

### 4.17. Statistical Analysis

Statistical significance was calculated with two-tailed Chi-square tests, ANOVA, and unpaired Mann–Whitney tests using InStat software (version 3.1, GraphPad, Los Angeles, CA, USA). Cox proportional hazard analysis was performed using the EZR program [54]. Survival curves were generated and statistical differences were calculated using the Kaplan–Meier method and log-rank test, respectively. Significance was defined as a two-sided *p*-value of <0.05.

## Figures and Tables

**Table 1:** Altered gene expression in MIA-GEM cells from MIA-P cells.

Increased	Fold Change (MIA-P = 1)
*MAST4*	7.1782656
*GJA1*	6.209089
*PDE1A*	6.1802254
*FLI1*	6.1545568
*ELOVL6*	6.0397415
*MMP1*	5.802817
*SYTL4*	5.648421
*ADD3*	5.6095138
*RHOBTB3*	5.5603056
*MAP3K5*	5.4986305
**Decreased**	**Fold Change (MIA-P = 1)**
*IFI16*	−8.182869
*ATPBD4*	−5.3832583
*PTPRZ1*	−5.1480074
*ACSL6*	-5.0153513
*AQP4*	−4.8476763
*COL1A1*	−4.837364
*GIT2*	−4.766745
*LOC644242*	−4.5996995
*LPHN3*	−4.380941
*SFRP1*	−4.3634024
*WDR26*	−4.1247654

**Table 2:** Nuclear *MAST4* expression in 91 PDAC cases.

Parameter			Nuclear *MAST4*	
		n	Index (%) ^1^	*p*-Value ^2^
Sex	Male	54	45 ± 15	
	Female	37	54 ± 20	NS
Age	23–52 y	46	48 ± 18	
	52–78 y	45	49 ± 17	NS
PS ^3^	1	32	48 ± 19	
	2	59	49 ± 22	NS
Grade ^4^	G1	31	28 ± 12	
	G2	29	48 ± 18	
	G3	31	68 ± 13	<0.0001
T factor ^4^	pT1-2	28	37 ± 14	
	pT3-4	63	54 ± 19	<0.0001
N factor ^4^	pN0	79	46 ± 18	
	pN1-2	12	68 ± 21	0.0002
Stage ^4^	I	26	17 ± 14	
	II	51	24 ± 18	
	III–IV	14	73 ± 23	<0.0001
RECIST ^5^	CR	2	15 ± 18	
	PR	15	18 ± 14	<0.0001
	SD	10	26 ± 15	
	PD	64	92 ± 17	<0.0001
GEM alone ^6^	CR/PR/SD	21	23 ± 13	
	PD	22	92 ± 16	<0.0001

^1^ Mean ± SD; ^2^ Statistical difference was calculated using Student’s *t*-test; ^3^ PS, performance status. ^4^ Clinicopathological parameters were described according to TNM classification: G1, well differentiated; G2, moderately differentiated; G3, poorly differentiated; T factor, local progression of primary tumor; pT1, tumor within the pancreas (<2 cm); pT2, tumor within the pancreas (2–4 cm); pT3, tumor still within the pancreas (>4 cm); pT4, tumor invading outside the pancreas; N factor, lymph node metastasis; pN0, no nodal metastasis; pN1-2, nodal metastasis to 1–4 or more nodes; Stage I–II, pT1-3/pN0-1; stage III, pT4/pNany; stage IV, pTany/pNany/M1. ^5^ RECST, responses evaluation criteria in solid tumor six months after chemotherapy., CR, complete response; PR, partial response; SD, stable disease; PD, progressive disease. ^6^ 43 patients received GEM alone.

**Table 3:** Relationship of nuclear *MAST4* expression with survival in 27 PDAC cases.

Parameter		MAST4-H ^1^	MAST4-L ^1^	*p*-Value ^2^
n		14	13	
Nuclear *MAST4* (%)		68 (48–81)	37 (14–47)	<0.0001
Sex	Male	7	7	
	Female	7	6	NS
Age	<52 y	5	5	
	<53 y	9	8	NS
PS ^3^	1	6	6	
	2	8	7	NS
Grade ^4^	G1	4	4	
	G2	5	5	
	G3	5	4	NS
T factor ^4^	pT1-2	12	11	
	pT3-4	2	2	NS
N factor ^4^	pN0	8	6	
	pN1-2	6	7	NS
Stage ^4^	I	0	1	
	II	9	7	
	III–IV	5	5	NS
RECIST ^5^	CR	0	0	
	PR	0	3	
	SD	3	6	
	PD	11	4	0.0268

^1^ The 27 cases were divided into two groups based on nuclear *MAST4* expression greater than the median value (MAST4-H) or less than the median value (MAST4-L). ^2^ Statistical difference was calculated using the Fisher’s exact test or Chi-square test; ^3^ PS, performance status. ^4^ Clinicopathological parameters were described according to TNM classification: G1, well differentiated; G2, moderately differentiated; G3, poorly differentiated; T factor, local progression of primary tumor; pT1, tumor within the pancreas (<2 cm); pT2, tumor within the pancreas (2–4 cm); pT3, tumor still within the pancreas (>4 cm); pT4, tumor invading outside the pancreas; N factor, lymph node metastasis; pN0, no nodal metastasis; pN1-2, nodal metastasis to 1–4 or more nodes; Stage I–II, pT1-3/pN0-1; stage III, pT4/pNany; stage IV, pTany/pNany/M1. ^5^ RECST, responses evaluation criteria in solid tumor six months after chemotherapy., CR, complete response; PR, partial response; SD, stable disease; PD, progressive disease.

**Table 4:** Multivariate analysis.

Parameters	Hazard Ratio	95% Confidential Interval	*p*-Value
T factor	0.06668	0.005297–0.8393	0.03612
N factor	0.18460	0.024960–1.3650	0.09786
Stage	4.24400	1.159000–15.5400	0.02904
*MAST4*	91.06000	2.837000–2923.0000	0.01080

*p*-value was calculated by Cox proportional hazard model using the EZR program.

**Table 5:** Primer sets, antisense, antibodies and ELISA kits.

Primer Set			
Gene Symbol	Gene Bank ID	Forward Primer (5′-3′)	Reverse Primer (5′-3′)
MAST4	NM_015183.2	ttccccaactggaatctgag	aggtggtgcttttggttttg
AKT1	KR710120.1	gcaccttccatgtggagact	cccagcagcttcaggtactc
AKT2	M95936.1	gaggtcatggagcacaggtt	ctggtccagctccagtaagc
AKT3	AJ245709.1	cagtagactggtggggccta	atcaagagccctgaaagcaa
BIM	AY352518.1	gcccctacctccctacagac	atggtggtggccatacaaat
PUMA	AF354654.1	ggagcagcacctggagtc	tactgtgcgttgaggtcgtc
FLIP	AB038972.2	cttggccaatttgcctgtat	tctttggcttccctgctaga
BCL2	M13994.1	acgacaaccgggagatagtg	catcccagcctccgttatcc
NOTCH1	CR457221.1	gatgtgtggactgtggcact	tgtgttgctggagcatcttc
NOTCH3	U97669.1	tgtggacgagtgctctatcg	aatgtccacctcgcaatagg
β-actin	NM_001101.3	ggacttcgagcaagagatgg	agcactgtgttggcgtacag
miR-582-5p	NR_030308.1	tgtgctctttgattacagttgttc	caccctttgggttcagttgt
Antisense			
MAST4	AS	aatgctggactcatccat	
	Control	nnnnnnnnnnnnnnnnnn	
**Antibody**			
**Protein**	**Clone**	**Company**	
E-cadherin	EP700Y	Abcam, Cambridge, MA, USA	
SNAIL	L70G2	Biocompare. South San Francisco, CA, USA	
CD44	F-4	Santa Cruz, Santa Cruz, CA, USA	
CD24	ERP19925	Abcam, Cambridge, MA, USA	
NS	-	antibodies-online GmbH, Aachen, Germany	
MAST4	-	Merck, Tokyo, Japan	
AKT1	1F7E10	Abcam, Cambridge, MA, USA	
AKT2	EP1676	Abcam, Cambridge, MA, USA	
AKT3	EE-M14	Santa Cruz, Santa Cruz, CA, USA	
Lamin	-	Abcam, Cambridge, MA, USA	
FOXO3	-	Abcam, Cambridge, MA, USA	
pAKT3 (pY312)	-	Bio-Rad. Hercules, CA, USA	
pFOXO3 (pS322/S325)	-	Cusabio, Houston, TX, USA	
β-actin	-	Abcam, Cambridge, MA, USA	
**ELISA**			
**Protein**	**Catalog#**		
AKT1	LS-F68359	LS Bio, Shirley, MA, USA	
AKT2	ab208986	Abcam, Cambridge, MA, USA	
AKT3	EH6215	Fine Test, Boulder CO, USA	
pFoxO3	28755-1-AP	Proteintech, Rosemont, IL, USA	

*MAST4*, microtubule-associated serine/threonine kinase family member 4; BIM, BCL2 like 11; PUMA, p53 upregulated modulator of apoptosis; FLIP, Fas-associated death domain-like interleukin-1-converting enzyme-like inhibitory protein; BCL2, B-cell lymphoma 2; NS, nucleostemin.

## Data Availability

Data are contained within the article.

## Abbreviations

PDAC, pancreatic ductal adenocarcinoma; MIA-P, parental MIA-PaCa-2; MIA-GEM, gemcitabine-resistant MIA-PaCa-2; GEM, gemcitabine; NS, nucleostemin; MAST4, microtubule-associated serine/threonine kinase family member 4; 5FU, 5-fluorouracil; CDDP, cisplatin; GSK3β, glycogen synthase kinase 3β; FOXO3, forkhead box protein O3; BIM, BCL2 like 11; PUMA, p53 upregulated modulator of apoptosis; FLIP, Fas-associated death domain-like interleukin-1-converting enzyme-like inhibitory protein; BCL2, B cell lymphoma 2; PI3K, phosphatidylinositol-3 kinase; JNK, c-Jun N-terminal kinase.
